# Mini-LEDs with Diffuse Reflection Cavity Arrays and Quantum Dot Film for Thin, Large-Area, High-Luminance Flat Light Source

**DOI:** 10.3390/nano11092395

**Published:** 2021-09-14

**Authors:** Zhi Ting Ye, Yuan Heng Cheng, Ku Huan Liu, Kai Shiang Yang

**Affiliations:** 1 Department of Mechanical Engineering, Advanced Institute of Manufacturing with High-Tech Innovations, National Chung Cheng University, No. 168 University Road, Minxiong Township, Chiayi County 621301, Taiwan; g09421023@ccu.edu.tw; 2 EPOCH CHEMTRONICS Corp, Hsinchu County 302006, Taiwan; GuuhuannLiu@epoch-optic.com (K.H.L.); t102568060@ntut.org.tw (K.S.Y.)

**Keywords:** mini-light-emitting diode, uniformity, luminance, diffuse reflection cavity array, quantum dot film

## Abstract

Mini-light-emitting diodes (mini-LEDs) were combined with multiple three-dimensional (3D) diffuse reflection cavity arrays (DRCAs) to produce thin, large-area, high-brightness, flat light source modules. The curvature of the 3D free-form DRCA was optimized to control its light path; this increased the distance between light sources and reduced the number of light sources used. Experiments with a 12.3-inch prototype indicated that 216 mini-LEDs were required for a 6 mm optical mixing distance to achieve a thin, large-area surface with high brightness, uniformity, and color saturation of 23,044 cd/m^2^, 90.13%, and 119.2, respectively. This module can serve as the local dimming backlight in next generation automotive displays.

## 1. Introduction

Display technology has developed rapidly, starting in the second half of the 20th century, and various types of displays have become ubiquitous in daily life. For example, light-emitting diodes (LEDs) have replaced conventional cathode-ray tubes. LEDs afford advantages such as higher color performance, low energy consumption, and eco-friendliness, and therefore, they are the principal backlight source of liquid crystal displays (LCDs) [1,2,3,4]. However, because of the demand for high resolution, high color saturation, and pixel quality, technologies including organic LEDs (OLEDs), mini-LEDs, micro-LEDs, quantum dot LEDs (QD-LEDs), laser displays, and holographic displays have been developed to replace LCDs. Nevertheless, LCDs remain the standard in the market and are widely used in smartphones, tablet computers, televisions, and other display devices [5,6,7,8]. As mentioned, LED light sources are used as LCD backlight sources. According to the position of the light source, they are divided into direct-type and side-type backlight modules. Direct-type backlights have lower cost, higher optical efficiency, and straightforward design compared with side-type backlights, and therefore, they have become dominant in the large-sized LCD backlight module market [9,10,11]. Although LCDs feature advantages such as low power consumption, long service life, and low cost, their slow response time, low color saturation, and low photoelectric conversion efficiency (typically <10%) are currently their main drawbacks [12,13,14,15]. To improve color saturation, color conversion materials with high response speed and high color saturation can be used. Furthermore, RGB chips can be used to form a white light source to increase the display color gamut. However, because of the relatively low efficiency of red LED chips, increasing the current and output power shortens the life of the chip. Blue LEDs with a GaN base and the green phosphor β-SiAlON and red phosphor K_2_SiF_6_ are commonly used as a high color gamut space backlight [16,17,18].

Quantum dots (QDs) are a new color conversion material that have a narrower full width at half-maximum (FWHM); therefore, they have a wider color gamut and higher quantum efficiency. As a result, novel QD-LEDs are gradually being applied to LCD backlight modules [19,20,21,22]. In addition, there are many studies that provide related synthesis protocols of quantum dots [23,24].

The strong demand for high-end displays such as gaming monitors, car monitors, and notebook computers has driven an increased demand for high dynamic area contrast backlights (i.e., high dynamic range [HDR]). Conventional LCD edge-lit backlights are only distributed on the light guide plate. Whether single- or double-sided, this architecture allows limited contrast control. Mini-LEDs, because of their smaller size and high current density injection drive, can achieve high-contrast HDR and high brightness. Mini-LEDs are thus considered a promising backlight source for next-generation high-end LCDs [25,26,27].

Mini-LEDs have a smaller package size than conventional LEDs. The light-emitting angle is limited to approximately 120°; therefore, more LEDs are required to achieve a uniform surface light source. Furthermore, the light line is not adjusted for HDR backlights, resulting in a halo. This problem causes the contrast to decrease, and the smaller area of the light source body causes the problem of heat source concentration [28,29,30,31].

Although the thickness of the backlight module can be reduced and the uniformity can be improved by increasing the number of mini-LEDs, this method is not the preferred design. Researchers have optimized the light output angle to reduce the total number of particles in the light source by adjusting the optical design [32,33]. For example, Ye et al. proposed a hollow tube structure containing mini-LEDs with asymmetrical luminous intensity and designed a beveled reflective surface on the side of the mini-LEDs to achieve high efficiency and high uniformity. Moreover, they proposed a flat light source module without a light guide plate with a dot pattern and a completely printed diffuse reflection light guide plate on the bottom surface of the light source module to achieve high efficiency and high uniformity [34,35]. Ohno proposed an LED lighting module with a coaxial light conductor to achieve high efficiency and high wide-angle performance [36]. Lu et al. proposed a transparent array of mini-LEDs on a polyethylene terephthalate (PET) substrate. They found that, at room temperature, the mini-LEDs exhibited high uniformity and wideness and stable color gamut performance. Lin et al. proposed a direct-illuminated LED free-form lens array with a Cartesian candela distribution. Through the incorporation of free lenses and diffusers, the LED lens array was optimized to improve uniformity. Zhu et al. proposed a free-form surface design method for LEDs to achieve diffusion and transmission. This lighting system affords advantages such as high efficiency and high uniformity [37,38,39].

Huang et al. proposed a chip-level package with a free-form package design that has ultrahigh light extraction efficiency and a batwing-shaped light field to provide a thin, high-wide-angle and low-profile package for reducing the number of LEDs. Li et al. proposed a two-layer encapsulation structure that combines SiO_2_ and graphite nanoparticles to improve the uniformity and environmental contrast. Chen et al. proposed a direct lighting mini-chip-level packaged LED that combines a QD film with a diffuser and prism films to improve the brightness and uniformity and to reduce the number of LEDs used [40,41,42].

Park et al. proposed a flip-chip package and a vertical chip package structure for InGaN/GaN stripe LEDs to achieve higher uniformity and to improve the light extraction efficiency. Tang et al. proposed the use of tetramethylammonium hydroxide to construct a layered structure on the sidewall of mini-LEDs by anisotropic etching to improve the light extraction efficiency of the mini-LEDs [43,44].

Zhu et al. proposed double freeform surface lens with diffuse reflection applied in non-imaging optical systems [45]. Zhu et al. proposed diffuse reflective Off-Axis surface for noncircular LED arrays [46]. Although these two designs can achieve large-area light sources, they cannot achieve a thin, high-luminance, flat light source. Sho et al. proposed a thin mini-LEDs backlight using reflective mirror dots to obtain high luminance uniformity for mobile LCDs [47].

In summary, related studies have attempted to improve the light output efficiency, uniformity, and color saturation of planar light sources. However, the use of large-area planar light sources to reduce the number of LEDs used requires further study.

This study proposes mini-LEDs combined with multiple three-dimensional (3D) diffuse reflection cavity arrays (DRCAs) to achieve a thin, large-area, and high-luminance planar light source.

## 2. Materials and Method

### 2.1. Simulation of Optical Module and Light Film Material Selection for Mini-LEDs

**Figure 1:**
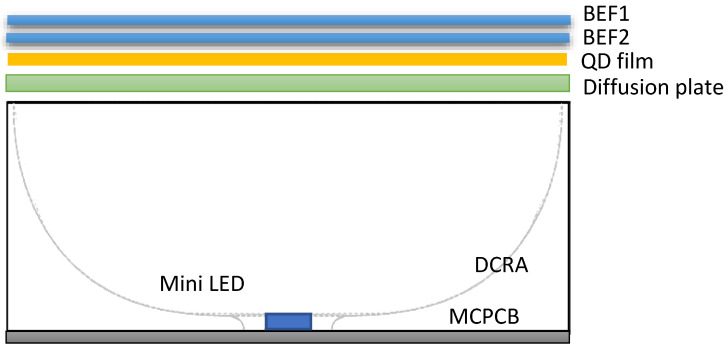
Schematic of the structure of mini-LEDs combined with 3D DRCA.

Soliwork3D drawing software and Light Tools optical simulation software were used to construct the optical system and optimize the diffuse reflection cavity module. The optical components include multiple 3D DRCAs, diffusers, QD films, and a brightness enhancement film (BEF). The influence of brightness and uniformity was analyzed. Figure 1 presents a schematic of the structure of the mini-LEDs combined with the 3D DRCA.

**Figure 2:**
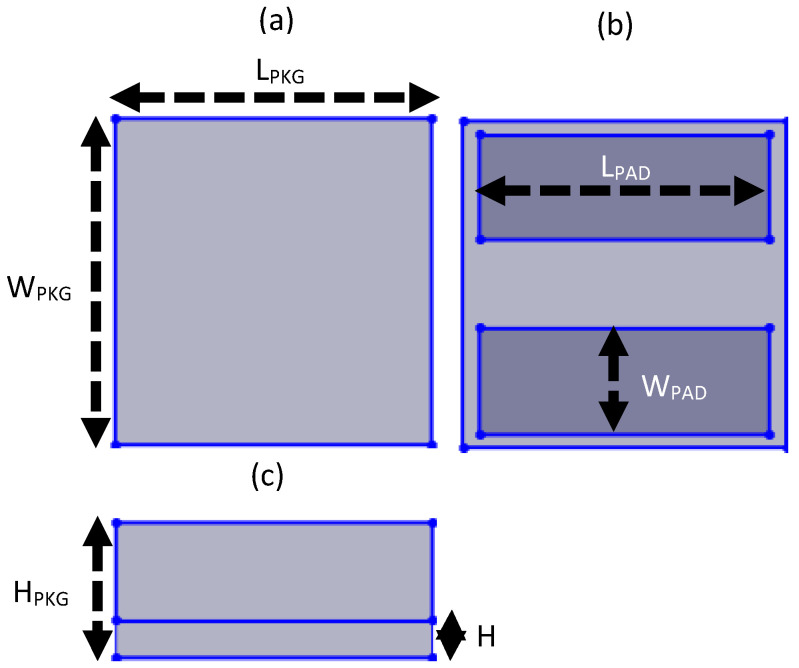
Structure of mini-LEDs. (**a**) top view, (**b**) bottom view, and (**c**) side view.

The light source uses Epistar YR-CV1010BDAN mini-LEDs. The package length L_PKG_, package width W_PKG_, package height H_PKG_, chip PAD length L_PAD_, and chip PAD width W_PAD_ are 1 mm, 1 mm, 0.4 m, 0.903 mm, and 0.328 mm, respectively, and Figure 2 displays the package size of the mini-LEDs.

**Figure 3:**
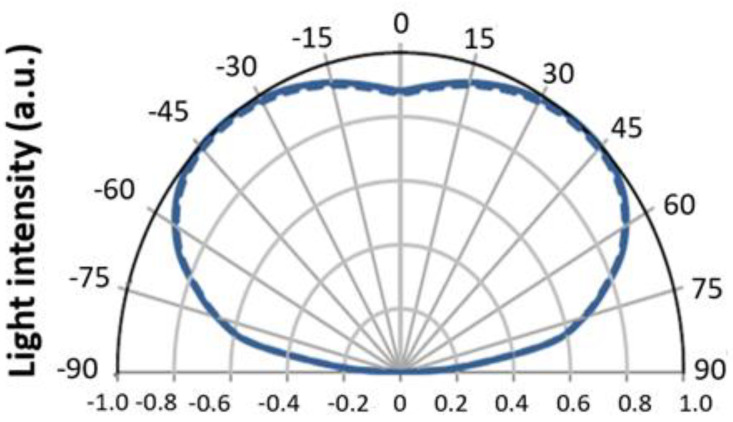
Mini-LED chip light distribution curve.

Figure 3 illustrates the light distribution curve of the mini-LEDs. The half-intensity angle FWHM is 160°, and the 0° angle intensity at the center is approximately 90%.

### 2.2. Model Construction of DRCA

A Bezier curve (B) was used to analyze the optimal curvature for the DRCA, as given by Equation (1).

(1) $$B\left(t\right)={\left(1-t\right)}^{3}P_{a}+3{\left(1-t\right)}^{2}tP_{b}+3\left(1-t\right)t^{2}P_{c}+t^{3}P_{d},\;t\;\in \;[0,\;1]\;$$

where the four points *P_a_*, *P_b_*, *P_c_*, and *P_d_* define a cubic Bezier curve in a plane or in a 3D space. The curve goes from *P_a_* to *P_b_* in the direction from *P_c_* to *P_d_*. Generally, it does not pass through *P_b_* or *P_c_*; these two points provide direction information. The distance between *P_a_* and *P_b_* determines the length of the curve in the direction of *P_b_* before turning to *P_c_*.

The equation of the Bezier curve is a continuous function; therefore, its derivative is obtained as follows:

(2) $$B’\left(t\right)=3tC_{1}+2tC_{2}+C_{3}$$

$C_{1}$, $C_{2}$, and $C_{3}$ are coefficients that are given as follows:

(3) $$C_{1}=(-P_{a}+3P_{b}-3P_{c}+P_{d}),\;C_{2}=(3P_{a}-6P_{b}+3P_{c}),\;\mathrm{and}\;C_{3}=(-3P_{a}+3P_{b}D)=P_{a}$$

**Figure 4:**
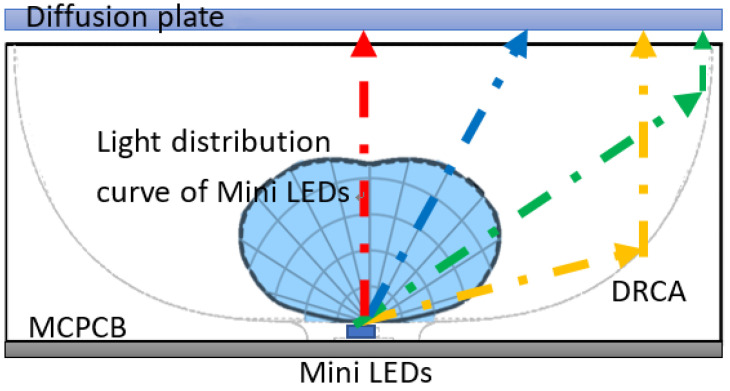
Schematic of diffuse reflection cavity light trace.

The light field shape of the mini-LEDs was used to simulate the diffuse reflection cavity, and the light was evenly distributed to the diffuser through an optimized design, thereby evenly expanding the projection area of the light source. Figure 4 presents a schematic of the light trace.

The parameters of the diffuse reflection cavity were set to the Lambertian diffuse surface characteristics: the diffuse reflectance was 94%, QD film was set to the Lambertian diffuse transmittance of 50%, reflectance was 50%, refractive index of the BEF was 1.56, and apex angle was 90°. The surface of the metal core printed circuit board (MCPCB) was set to the Lambertian diffuse surface reflectance of 90%, center wavelength of the light source was 450 nm, input power was normalized as 1 W, and number of rays was 50 million for simulation. Table 1 lists the parameter settings.

**Figure 5:**
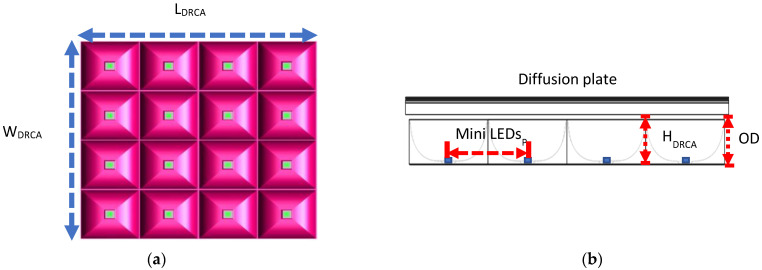
Schematic of the 3D structure of the 4 × 4 diffuse reflection cavity matrix modules. (**a**) top view and (**b**) side view.

L_DRCA_, W_DRCA_, and H_DRCA_ of the simulated diffuse reflection cavity matrix module were 48.8, 48.72, and 4.57 mm, respectively. Furthermore, the mini-LED spacing mini-LEDs_p_ was 12 mm, optical mixing distance between the mini-LEDs and the diffuser was 6 mm, and the simulation of the design optimization was based on a 4 × 4 diffuse reflection cavity matrix. Figure 5 illustrates the 3D structure of the module.

### 2.3. Fabrication of QD Film

Common QD film materials, such as green QDs (λg: ~530 nm) and red QDs (λg: ~626 nm) (both from Unique Materials Co. Ltd., Taichung city, Taiwan), were used. The quantum dots used in this article are red and green CdSe/ZnS. The particle size ranges of green and red quantum dots are 2.5–2.8 nm and 4.4–4.6 nm, respectively. The main reaction materials are cadmium oxide (CdO), zinc oxide (ZnO), silicon (Se) and Trioctylphosphine oxide (TOPO), lauric acid (C_12_H_24_O_2_), and n-hexane (C_6_H_14_) material match reaction. Hexadecyl amine (HDA) was used to prevent the agglomeration reaction of QDs; methanol (CH_3_OH) was used to avoid agglomeration reaction, and argon (Ar) was used throughout the process to ensure inert atmosphere while growing the QDs.

The produced QDs were cured in Poly(methyl methacrylate) (PMMA); the weight ratio of quantum dots and PMMA glue is 20:1, and the weight percent concentration is 5 wt.%. The volume ratio of the red quantum-dots (QDs) and green QDs is 1:50.

**Figure 6:**
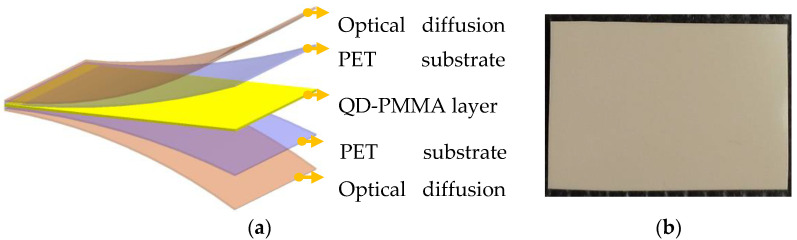
Structure of QD color conversion film. (**a**) Various layers in structure and (**b**) actual sample.

The QD-PMMA layer was coated between two PETS film layers. Subsequently, the PET/QD-PMMA/PET film was laminated and cured using 365 nm ultraviolet radiation. Finally, a light diffusion layer was coated on the PET/QD-PMMA/PET film with a doctor blade coater. Figure 6 displays the structure.

### 2.4. Simulation and Optimization of DRCA Structure

**Figure 7:**
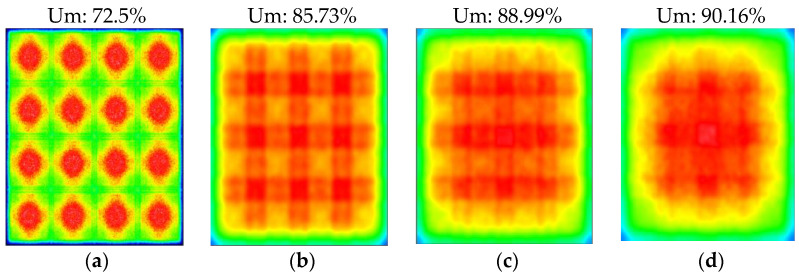
Simulation and optimization results of mini-LEDs combined with the diffuse reflection cavity matrix. (**a**) Mini-LEDs combined with diffuse reflection cavity matrix, (**b**) additional diffuser, (**c**) additional diffuser and first BEF, and (**d**) additional diffuser and second BEF.

Figure 7 presents the simulation result. First, a simulation was performed to optimize the diffuse reflection cavity matrix. Subsequently, the diffuser and BEF were added in sequence, and their influence on the uniformity of the light was analyzed. The uniformity was calculated as Min-luminance/Max-luminance × 100%. Figure 7a reveals the optimized diffuse reflection cavity matrix. The mini-LED point light source was converted into a character with a wider light output area and uniformity of 72.5%. As indicated in Figure 7b, the diffuse reflection cavity matrix was projected onto an added diffuser to further expand the projection area and improve the uniformity to 85.73%. Figure 7c shows the addition of the first piece of the BEF. The simulation results indicated that adjusting the angle of light helped improve the uniformity to 88.99%. Finally, Figure 7d shows the addition of the first two pieces of BEF to adjust the concentrated light angle again and achieve uniformity of 90.16%.

## 3. Results and Discussion

**Figure 8:**
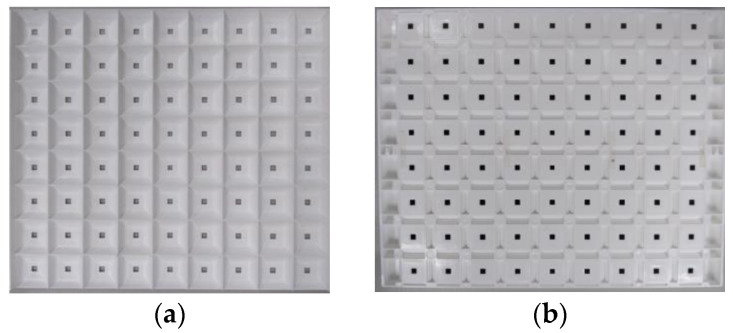
Actual sample of 9 × 8 DRCA. (**a**) Front view and (**b**) back view of the diffuse reflection cavity.

Figure 8 presents the front and back of the actual sample of the 9 × 8 DRCA. The square transparent part in the figure is the diffuse reflection cavity, and the gray and black points in the center of the square are mini-LEDs.

**Figure 9:**
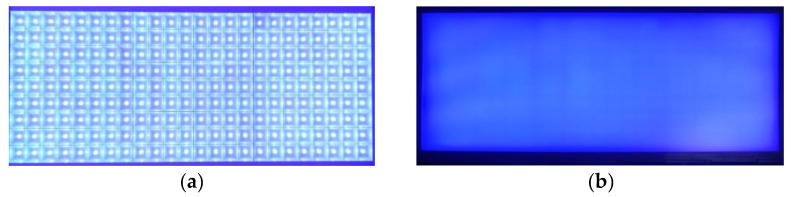
Actual sample of a 12.3-inch thin blue large-area high-brightness flat light source module. (**a**) mini-LEDs combined with DRCA and (**b**) mini-LEDs combined with DRCA and diffuser.

Figure 9 depicts the actual sample of the 12.3-inch thin, large-area, high-brightness flat light source module. It comprises three groups of 9 × 8 DRCAs. The length, width, and height of the module are 292.8, 121, and 4.57 mm, respectively; the optical mixing distance is 6 mm, number of mini-LEDs is 216, and dynamic designable area comprises 216 zones.

**Figure 10:**
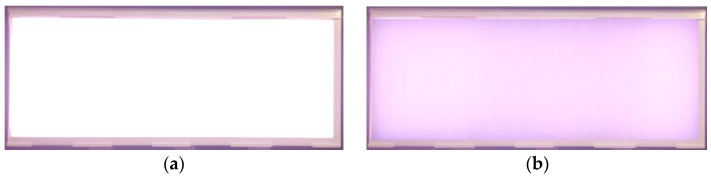
Actual sample diagram of 12.3-inch white light thin large-area high-brightness flat light source module, (**a**) mini-LEDs combined QD color conversion film with DRCAs and (**b**) mini-LEDs combined QD color conversion film without DRCAs.

Figure 10 shows the actual sample diagram of the 12.3-inch white light thin large-area high-brightness flat light source module. Figure 10a shows the light source module that comprises 216 wide-angle mini-LEDs combined with DRCAs and QD color conversion film. Figure 10b shows mini-LEDs combined QD color conversion film without DRCAs. The driving conditions are a forward voltage of 12 V and forward current of 1.65 A.

**Figure 11:**
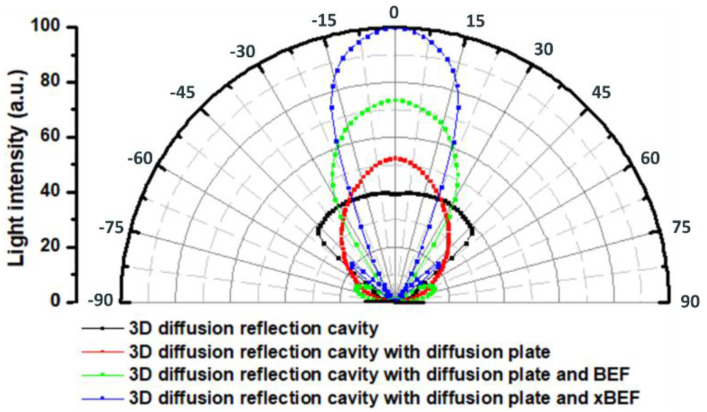
Light distribution curve of a 12.3-inch planar light source module with different structures.

Figure 11 presents the normalized light distribution curve obtained through an actual measurement. The black line indicates the light distribution curve of mini-LEDs combined with a DRCA. In this case, the central intensity value was 39.34%. The red line indicates the light distribution curve of mini-LEDs combined with a DRCA and a diffuser. In this case, the central intensity value increased to 52.42%. The green line indicates the light distribution curve of mini-LEDs combined with a DRCA, a diffuser, and the first BEF. In this case, the central intensity value increased to 66.88%. The blue line indicates the light distribution curve of the mini-LEDs combined with a DRCA, a diffuser, and the second BEF. In this case, the central intensity value increased to 100%. These results indicate that for mini-LEDs combined with a DRCA as well as the first and the second BEF, the central light intensity can be increased by 1.7 and 2.54 times, respectively. The difference in light intensity between the two centers was 1.494 times.

**Figure 12:**
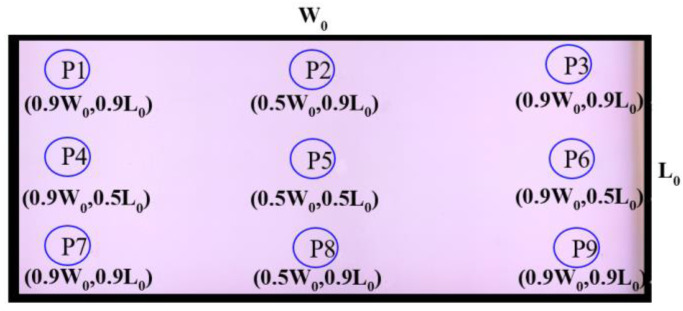
Brightness measurement points for the 12.3-inch white light thin large-area high-brightness flat light source module.

The uniformity of the 12.3-inch white light thin large-area high-brightness planar light source module was measured using the nine-point measurement method commonly used in the industry. Figure 12 presents the measurement point distribution.

The uniformity was calculated as follows:

(4) $$\mathrm{Uniformity}\;(\%)=100\%\;\times \;\frac{minimum\;luminance\;\left(nits\right)}{maximum\;luminance\;\left(nits\right)}$$

Table 2 lists the brightness value measured at nine measuring points and the uniformity value of the 12.3-inch mini-LEDs combined QD color conversion film with DRCAs of planar light source module. The driving conditions are a forward voltage of 12 V and forward current of 1.65 A. On average, the uniformity of the module was 87.58% when one BEF was added, and the brightness was 15,240 cd/m^2^. Furthermore, the uniformity of the module was 90.13% when two BEFs were added, and the average brightness was 23,044 cd/m^2^. Therefore, the difference in average brightness between the two was 1.51 times. The average luminance value measured and the uniformity value of general mini-LED backlight that mini-LEDs combined QD color conversion film without DRCAs is 19,370 cd/m^2^ and 74.89%, respectively. Comparison of mini-LEDs backlight combined with DRCAs and without DRCAs shows that luminance is enhanced 1.18 times, and uniformity increases from 74.89% to 90.13%.

**Figure 13:**
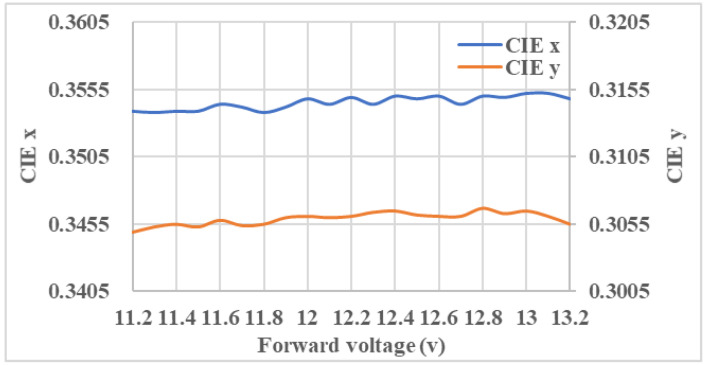
The measured CIE color coordinates (x,y)–voltage of wide-angle mini-LEDs combined with a DRCA and QD film light source module.

Figure 13 displays the measured CIE color coordinates(x,y)-voltage of wide-angle mini-LEDs combined with a DRCA and QD film light source module. The forward voltage is increased from 11.2 V to 13.2 V, and the maximum variations in CIE x,y are 0.0014 and 0.0018, respectively.

**Figure 14:**
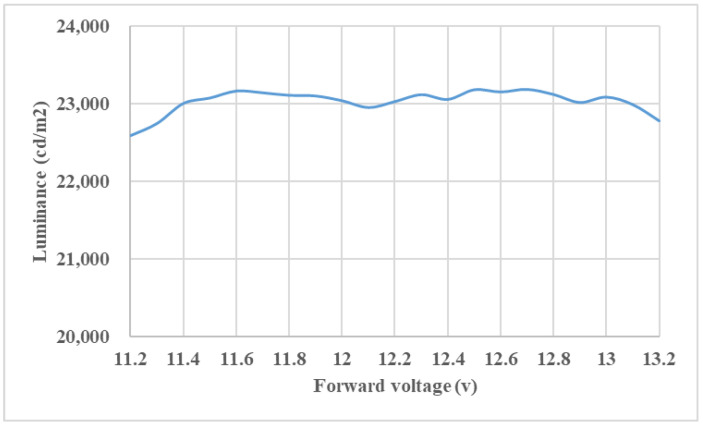
Displays the measured luminance (cd/m^2^)–voltage of wide-angle mini-LEDs combined with a DRCA and QD film light source module.

Figure 14 displays the measured luminance (cd/m^2^)–voltage of wide-angle mini-LEDs combined with a DRCA and QD film light source module. The forward voltage is increased from 11.2 V to 13.2 V, the maximum variation percentage of luminance is 2.6%.

**Figure 15:**
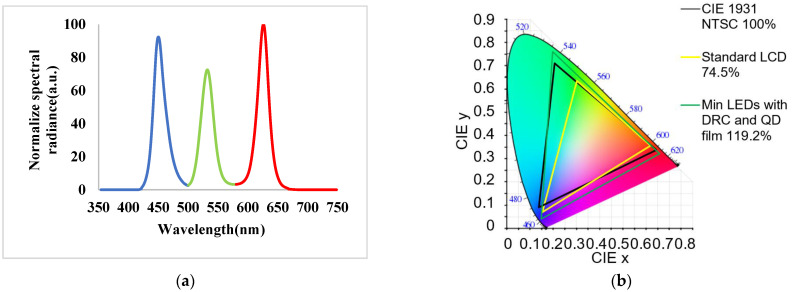
(**a**) Normalized EL spectrum and (**b**) color gamut of mini-LEDs combined with a DRCA and QD film optical module.

Figure 15 displays the electroluminescence (EL) spectrum of wide-angle mini-LEDs combined with a DRCA and QD film light source module. In Figure 15a, the peak emission wavelengths are 450, 532, and 626 nm, and the FWHM is 20 nm. These results indicate that the mini-LEDs have the advantage of high color purity. According to the EL spectrum in the CIE 1931 color gamut coordinates, the mini-LEDs’ NTSC reached 119.2%, as shown in Figure 15b, indicating their wide color gamut range.

## 4. Conclusions

This study optimized the design of mini-LEDs combined with multiple 3D DRCAs for application to thin, large-area, high-brightness, flat light source modules. A 12.3-inch white light thin, large-area, high-brightness planar light source module was used as a sample. The driving condition is a forward voltage of 12 V and a forward current of 1.65 A. At a mixing distance of 6 mm, the average luminance reached 23,044 cd/m^2^, and NTSC was 119.2%. Comparison of mini-LEDs backlight combined with DRCAs to that without DRCAs shows that luminance is enhanced 1.18 times, and uniformity increases from 74.89% to 90.13%. The thin, large-area, high-brightness, high-uniformity, high-color-saturation planar light source module proposed in this study can be employed as the local dimming backlight in next-generation automotive displays.

## Figures and Tables

**Table 1:** Simulation parameter settings.

BEF1	The refractive index = 1.56, the apex angle = 90 degrees
BEF2	The refractive index = 1.56, the apex angle = 90 degrees
diffuse reflection cavity array, DRCA	Lambertian diffusion surface characteristics, Reflectance 94%
QD film	Lambertian diffusion surface characteristics, 50% transmittance and 50% reflectivity
MCPCB	Lambertian diffusion surface characteristics, Reflectance 90%
Light source	Input light 1 W Center wavelength 450 nm 50 million rays

**Table 2:** Brightness and uniformity measured at nine points on the actual sample.

	1 BEF Luminance (cd/m^2^)	2 BEF Luminance (cd/m^2^)
P1	15,910	23,695
P2	14,220	21,785
P3	15,730	23,758
P4	16,218	24,025
P5	14,335	21,884
P6	15,276	23,255
P7	15,832	23,758
P8	14,205	21,655
P9	15,430	23,585
Average luminance/ CIE (x,y)	15,240/ CIE (x 0.3268,y 0.2845)	230,44/ CIE (x 0.3545,y 0.3059)
Uniformity (%)	87.58%	90.13%

## Data Availability

Data sharing is not available.
